# Iminosugars Inhibit Dengue Virus Production via Inhibition of ER Alpha-Glucosidases—Not Glycolipid Processing Enzymes

**DOI:** 10.1371/journal.pntd.0004524

**Published:** 2016-03-14

**Authors:** Andrew C. Sayce, Dominic S. Alonzi, Sarah S. Killingbeck, Beatrice E. Tyrrell, Michelle L. Hill, Alessandro T. Caputo, Ren Iwaki, Kyoko Kinami, Daisuke Ide, J. L. Kiappes, P. Robert Beatty, Atsushi Kato, Eva Harris, Raymond A. Dwek, Joanna L. Miller, Nicole Zitzmann

**Affiliations:** 1 Oxford Glycobiology Institute, Department of Biochemistry, University of Oxford, Oxford, United Kingdom; 2 Division of Infectious Diseases and Vaccinology, School of Public Health, University of California-Berkeley, Berkeley, California, United States of America; 3 Department of Hospital Pharmacy, University of Toyama, Toyama, Japan; University of Texas Medical Branch, UNITED STATES

## Abstract

It has long been thought that iminosugar antiviral activity is a function of inhibition of endoplasmic reticulum-resident α-glucosidases, and on this basis, many iminosugars have been investigated as therapeutic agents for treatment of infection by a diverse spectrum of viruses, including dengue virus (DENV). However, iminosugars are glycomimetics possessing a nitrogen atom in place of the endocyclic oxygen atom, and the ubiquity of glycans in host metabolism suggests that multiple pathways can be targeted via iminosugar treatment. Successful treatment of patients with glycolipid processing defects using iminosugars highlights the clinical exploitation of iminosugar inhibition of enzymes other than ER α-glucosidases. Evidence correlating antiviral activity with successful inhibition of ER glucosidases together with the exclusion of alternative mechanisms of action of iminosugars in the context of DENV infection is limited. Celgosivir, a bicyclic iminosugar evaluated in phase Ib clinical trials as a therapeutic for the treatment of DENV infection, was confirmed to be antiviral in a lethal mouse model of antibody-enhanced DENV infection. In this study we provide the first evidence of the antiviral activity of celgosivir in primary human macrophages *in vitro*, in which it inhibits DENV secretion with an EC_50_ of 5 μM. We further demonstrate that monocyclic glucose-mimicking iminosugars inhibit isolated glycoprotein and glycolipid processing enzymes and that this inhibition also occurs in primary cells treated with these drugs. By comparison to bicyclic glucose-mimicking iminosugars which inhibit glycoprotein processing but do not inhibit glycolipid processing and galactose-mimicking iminosugars which do not inhibit glycoprotein processing but do inhibit glycolipid processing, we demonstrate that inhibition of endoplasmic reticulum-resident α-glucosidases, not glycolipid processing, is responsible for iminosugar antiviral activity against DENV. Our data suggest that inhibition of ER α-glucosidases prevents release of virus and is the primary antiviral mechanism of action of iminosugars against DENV.

## Author Summary

Current treatment of dengue virus infection is supportive; however, iminosugars have been widely investigated as an antiviral strategy. The means by which these molecules are thought to exert their antiviral effects is through inhibition of host-resident glycoprotein processing enzymes, the endoplasmic reticulum-resident α-glucosidases, but many iminosugars are also capable of inhibiting host glycolipid processing and are utilized clinically for the treatment of lysosomal storage disorders such as Gaucher’s and Niemann-Pick type C diseases. The work presented here is the first to conclusively differentiate the antiviral properties of these two major mechanisms of action of iminosugars, and our data support the long-standing hypothesis that inhibition of glycoprotein processing is the essential antiviral property of iminosugars in the case of dengue virus infection. These results indicate that further development of iminosugars as dengue antivirals should focus on optimization of glycoprotein inhibition efficacy with reduction or elimination of glycolipid modulating properties to minimize off-target effects. These results are supported by the *in vitro* and *in vivo* efficacy of the bicyclic iminosugar, celgosivir, which we demonstrate to lack capacity for inhibition of glycosphingolipid processing.

## Introduction

Iminosugars are considered to be promising candidates for broad-spectrum antiviral activity because of their presumed mechanism of action as glycoprotein processing inhibitors [1]. 1-Deoxynojirimycin (DNJ) iminosugar derivatives possess glucose stereochemistry and inhibit infectious virus production *in vitro* of viruses including dengue virus (DENV) [2–7], hepatitis B virus (HBV) [8,9], hepatitis C virus (HCV) [10], human immunodeficiency virus (HIV) [11], and influenza A virus [12]. Bicyclic iminosugars possessing glucostereochemistry, such as castanospermine, also inhibit infectious virus production *in vitro* [11,13–15]. Antiviral efficacy of both bicylic and monocyclic iminosugars has been further demonstrated *in vivo*, particularly against DENV infection, with protection in lethal mouse models conferred by post-exposure therapeutic administration of *N*-butyl-DNJ (*N*B-DNJ, Miglustat, Zavesca) [5], *N*-(9-methoxynonyl)-DNJ (UV4, M*O*N-DNJ) [16], and 6-*O*-butanoyl-castanospermine (BuCAST, celgosivir) [17,18]. These promising *in vitro* and *in vivo* results have led to clinical trials of both M*O*N-DNJ and celgosivir as dengue therapeutics. The antiviral activity of iminosugars is presumed to be a function of inhibition of endoplasmic reticulum (ER)-resident α-glucosidases I and II; thus, it has been hypothesized that antiviral efficacy of this class of drugs may be refractory to development of drug-resistant mutants because a host protein, rather than a viral protein, is the target of drug activity. Therefore treatment should be relatively refractory to escape and be effective against many viruses possessing *N*-linked glycoproteins [1,19].

DENV possesses four *N*-linked glycoproteins: envelope, pre-membrane, non-structural protein 1, and non-structural protein 4B [20]. Previous work has demonstrated that iminosugar treatment reduces association of DENV glycoproteins with the ER-resident glycoprotein chaperone calnexin, and as a result, secretion of the three DENV glycoproteins studied (envelope, pre-membrane, and non-structural protein 1) was reduced [6]. Furthermore, siRNA-mediated reduction of calnexin, calreticulin, or human immunoglobulin heavy chain binding protein (BiP), which is another glycoprotein chaperone, led to decreased production of infectious DENV [21]. Generation of free oligosaccharides (FOS), which are markers of inhibition of ER α-glucosidase activity [22], has been correlated with iminosugar antiviral activity for two DNJ-derived iminosugars [23]. Although these reports present strong circumstantial evidence for dependence of iminosugar antiviral activity on inhibition of ER α-glucosidase activity, the ubiquity of d-glucose in metabolism suggests that other pathways may be equally affected by iminosugar treatment. Indeed, *N*B-DNJ has been approved for clinical use since 2002 as a treatment for Gaucher’s Disease [24]–a lysosomal storage disorder. In this context, *N*B-DNJ is used as an inhibitor of glucosylceramide synthase (GCS) to reduce production of glycosphingolipids (GSLs) that accumulate to the detriment of normal cellular function [25,26]. It has been suggested that neutral GSLs are of importance for binding of DENV to both mammalian and mosquito cell surfaces [27], and large-scale rearrangements of the endoplasmic reticulum and associated membranes during the course of DENV infection suggest that significant rearrangement of the cellular (glyco)lipid pool is necessary to support DENV infection and replication [28,29]. Thus, iminosugar antiviral activity may be dependent upon perturbation of host glycolipid processing rather than, or concomitant with, host glycoprotein processing. A clear understanding of the role of interference with glycolipid processing in iminosugar antiviral activity is essential for defining the mechanism(s) of action of this class of drug against DENV.

**Fig 1:**
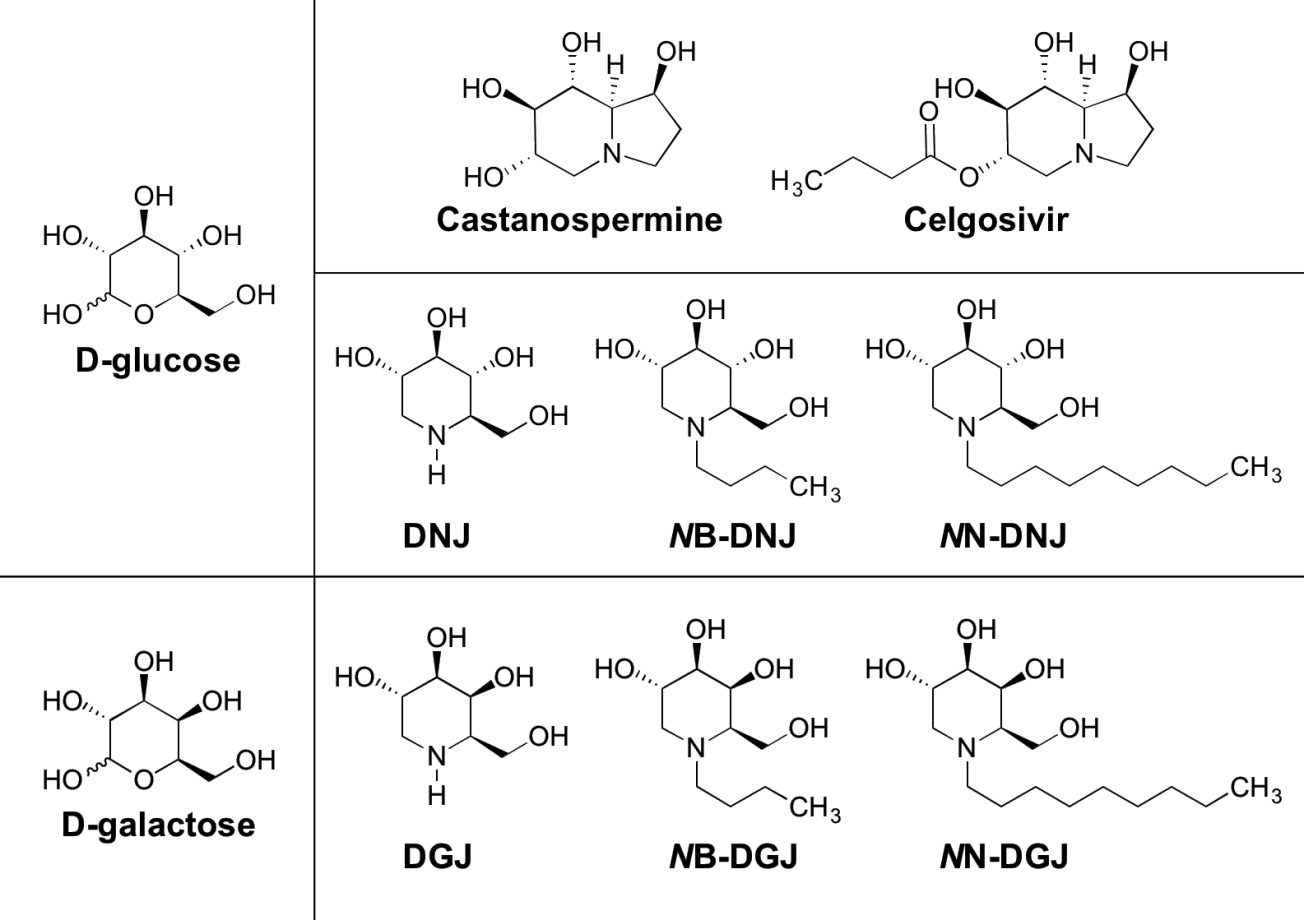
Iminosugars used in this study. Iminosugars are sugar mimics with nitrogen substitution of the endocyclic oxygen. Sugars from which iminosugars are derived are presented to the left with d-glucose on top (white background) and d-galactose on the bottom (grey background). A lead clinical candidate for DENV antiviral activity, celgosivir, is a pro-drug of castanospermine, both of which possess d-glucose stereochemistry. A series of deoxynojirimycin (DNJ) derivatives with variable alkylation of the ring nitrogen was synthesized for comparison to equivalent galactose mimic deoxygalactonojirimycin (DGJ) derivatives.

In this study, we delineate the relative antiviral contribution of glycoprotein and glycolipid modulation by iminosugars. In order to determine the role of glycolipid inhibition in iminosugar antiviral effect, we utilized a small panel of the most commonly investigated DNJ iminosugars and an equivalent panel of structurally similar 1-deoxygalactonojirimycin (DGJ) iminosugars (Fig 1). DGJ analogues are identical to their DNJ counterparts with the exception of the stereochemistry of the C4-hydroxyl group to achieve galactose, rather than glucose, stereochemistry of the sugar head-group. By comparison of *in vitro* and cellular enzyme inhibition profiles of DNJ and DGJ iminosugars, we determine the roles of iminosugar inhibition of glycolipid and glycoprotein processing on DENV antiviral activity.

## Methods

### Virus stocks

For *in vitro* infection assays, DENV2 strain 16681 (a gift from E. Gould, Centre for Ecology and Hydrology, Oxford, UK) was propagated in C6/36 *Aedes albopictus* cell line (US Armed Forces Research Institute of Medical Sciences, Thailand (AFRIMS)), collected from supernatant, and concentrated by precipitation with 10% weight per unit volume (w/v) poly(ethyleneglycol) M_r_ 6,000 (Sigma), 0.6% sodium chloride (Sigma) overnight at 4°C. Following precipitation, virus was centrifuged at 2830 x *g* for 45 minutes at 4°C, resuspended in Leibovitz’s L15 + 10% HI-FBS, and stored at –80°C until use. For *in vivo* experiments, mouse-adapted DENV2 strain D2S10 was amplified in C6/36 cells as described previously [30].

### Tissue culture

#### Isolation and differentiation of monocyte-derived macrophages (MDMΦs)

Human peripheral blood mononuclear cells (PBMCs) were isolated from buffy coats (NHS Blood and Transplant, surplus to clinical requirements) by centrifugation over a Ficoll-Paque PLUS (Amersham) gradient. Autologous plasma was collected, heat inactivated (56°C, 30 minutes), and used to supplement (1%) X-VIVO10 (Lonza) medium to produce MDMΦ growth medium. Adherent monocytes were isolated from PBMCs by incubation on 2% bovine gelatin (Sigma) coated tissue culture plates (37°C, 5% CO_2_, 90 minutes). Non-adherent PBMCs were washed off in RPMI-1640 (Sigma), and remaining monocytes were incubated in MDMΦ growth medium (37°C, 5% CO_2_, 18 hours). Supernatants at 18 hours post-seeding containing monocytes were collected, and additional monocytes were collected by mechanical removal (vigorous pipetting) following incubation in ice-cold sterile PBS + 5 mM EDTA (Sigma) (4°C, 90 minutes) and combined with monocytes in supernatant. Cells were seeded at assay-dependent densities (1–1.5 x 10^6^ cells/ml) in MDMΦ growth medium with 25 ng/ml recombinant human IL-4 (rhIL-4, Peprotech) and differentiated for 3 days (37°C, 5% CO_2_) to generate MDMΦs. Cells generated in this manner are greater than 95% macrophages phenotypically (CD14^+^, CD16^-^, CD86^+^, HLA-DR^+^, CD3^-^) by fluorescence activated cell sorting (FACS) as previously reported [31]. The use of human blood was approved by the NHS National Research Ethics Service (09/H0606/3).

#### Culture of cell lines

*Macaca mulatta* kidney cells (LLC-MK2, AFRIMS) were propagated in Medium 199 (Gibco) supplemented with 20% heat-inactivated foetal bovine serum (HI-FBS) (Seralab), 0.1 mg/ml streptomycin (Gibco), and 100 U/ml penicillin (Gibco) at 37°C, 5% CO_2_. *Aedes albopictus* C6/36 cells (AFRIMS) were maintained in Leibovitz’s L15 medium (Gibco) supplemented with 10% HI-FBS, 2 mM L-glutamine (Gibco), 0.1 mg/ml streptomycin (Gibco), and 100 U/ml penicillin (Gibco) at 28°C in unvented tissue culture flasks in the absence of DENV infection. During DENV infection of C6/36, supplements were diluted 3:20 to final concentrations of 1.5% HI-FBS, 300 μM L-glutamine, 15 μg/ml streptomycin, and 15 U/ml penicillin. Baby hamster kidney cells (BHK-21, ATCC) were propagated in α-MEM (Gibco) supplemented with 5% HI-FBS, 2 mM L-glutamine, 0.1 mg/ml streptomycin, and 100 U/ml penicillin at 37°C, 5% CO_2_.

### Iminosugars used in this study

The iminosugar compounds tested herein were synthesized according to previously reported protocols or obtained from industrial sources. Monocylic iminosugars include d-DNJ (solubilised in water), *N*B-DNJ (solubilised in PBS, gift from Oxford GlycoSciences Ltd.), *N*N-DNJ (solubilised in 83% dimethyl sulfoxide (DMSO)), d-DGJ (solubilised in water), *N*B-DGJ (solubilised in 83% DMSO, Toronto Research Chemicals) and *N*N-DGJ (solubilised in 83% DMSO, Toronto Research Chemicals). Bicyclic iminosugars include castanospermine (solubilised in water, Cambridge Biosciences Ltd) and celgosivir (solubilised in water, gift from Subhash Vasudevan).

### *In vitro* enzyme inhibition assays

Inhibition of glucosidases and glycosidases was detected as previously described [32]. Briefly, assays were performed at the optimum *p*H of each enzyme, and appropriate disaccharide was provided as substrate for glucosidase release assays. Release of d-glucose was measured using a Glucose B-test (Wako Diagnostics). Appropriate *p*-nitrophenyl glycoside was added as substrate for additional glycosidase assays, and assays were stopped by adding 400 mM Na_2_CO_3_. Release of *p*-nitrophenol was measured spectrophotometrically at 400 nm.

Recombinant *Mus musculus* ER alpha-glucosidase II inhibition was measured by incubating 20 nM enzyme with various concentrations of inhibitors in 100 mM potassium phosphate pH 7.2 at 37^°^C for 5 minutes. Activity was measured by addition of 0.25 mM 4-methylumbelliferyl α-d-glucopyranoside (4MUG) (Sigma Aldrich) in black, non-binding surface-treated microplates (Corning). Fluorescence upon hydrolysis of the 4MUG was measured at λ_ex_ of 355 nm and λ_em_ of 460 nm in a M5 multi-mode plate reader (Molecular Devices) at 37^°^C, measuring fluorescence every minute for a total of 30 minutes. The initial velocity was fit in the linear range of measurements, and activity was normalized to the uninhibited control in order to plot activity against the inhibitor concentration. A sigmoidal, four-parameter function with a variable slope was used to determine the EC_50_ values in Prism (GraphPad).

### Cytotoxicity

Cytotoxicity of compounds was assessed by a cell proliferation assay for metabolic activity using a CellTiter 96 AQ_ueous_ One Solution Cell Proliferation Assay (Promega) as per the manufacturer’s instructions. Briefly, 20 μl of a solution containing tetrazolium compound [3-(4,5-dimethyl-2-yl)-5-(3-carboxymethoxyphenyl)-2-(4-sulfophenyl)-2H-tetrazolium; MTS] and electron coupling reagent (phenazine ethosulfate) was added to cells covered or suspended in 100 μl of culture medium in a 96-well plate. Samples were incubated for approximately 4 hours (37°C, 5% CO_2_), and the absorbance at 490 nm (A_490_) was measured on a SpectraMax M5 microplate reader (Molecular Devices). Absorbance was normalised to untreated controls whereby cytotoxicity resulted in decreased A_490_.

### *In vitro* virus infection and drug treatment

MDMΦ were infected with DENV2 16681 diluted to a multiplicity of infection (MOI) of 1 in X-VIVO10 without supplements for 90 minutes (20°C, with rocking). Upon removal of virus, fresh MDMΦ growth medium without IL-4 but containing serial dilutions of drug or control was placed on the cells, and cells were incubated for 48 hours (37°C, 5% CO_2_). For collection of infectious virus, supernatant was harvested and centrifuged for 5 minutes (room temperature, 400 x *g*) to pellet any cells/debris, and supernatants were aliquoted and stored at -80°C until analysis by plaque assay. In the case of FOS and GSL assays where MDMΦ were uninfected, treatments were essentially as above except that no virus was added and cells were washed three times with sterile PBS after 24 or 48 hours as per sample time point. On the third wash, cells were removed from tissue culture plastic by mechanical disruption (scraping) and transferred to microcentrifuge tubes for centrifugation (room temperature, 4000 x *g*, 5 minutes). Cells were lysed by three cycles of freeze-thawing (alternating room temperature and –80°C) in deionised H_2_O and stored at –80°C for subsequent protein, FOS, or GSL assay.

### Plaque assays

Virus titres for samples from human MDMΦ and C6/36 infections were obtained by LLC-MK2 plaque assays as per previous descriptions [33]. Briefly, LLC-MK2s were seeded in confluent monolayers, allowed to adhere, and washed once with Hank’s balanced salt solution (HBSS, Gibco). Log_10_ serial dilution of viral supernatants was conducted in DMEM10 (Gibco), and virus was incubated on cells for 90 minutes (20°C, with rocking). Upon removal of virus, first-overlay containing nutrients and low-melting point tissue culture-grade agarose [33] was added and allowed to solidify at 20°C before incubation for 5 days (37°C, 5% CO_2_). A second-overlay containing nutrients, low-melting point tissue culture-grade agarose, and neutral red stain [33] was added and allowed to solidify at 20°C before further incubation for 1 day (37°C, 5% CO_2_) after which plaques were counted by eye. The limit of detection for this assay is 33 plaque forming units per ml (pfu/ml).

Virus titers of samples isolated from infected mice were obtained by BHK-21 plaque assays in accordance with previous protocols [15,34]. BHK-21 cells were seeded in 12-well plates (3 x 10^5^ cells/well) in α-MEM medium containing 5% HI-FBS for 3 hours (37°C, 5% CO_2_). Medium was removed, log_10_ serial dilutions of virus in α-MEM with 2% HI-FBS were added, and cells were incubated for an additional 2 hours (37°C, 5% CO_2_). Following incubation, 1.5 ml of α-MEM containing 5% (v/v) HI-FBS and 1% (w/v) low-melting point tissue culture grade agarose (Sigma) was added to each well and allowed to solidify at room temperature before further incubation (37°C, 5% CO_2_) for 5 days. Plaques were visualised after fixation in 10% formaldehyde (1 hour, room temperature) and removal of the agarose plug by staining briefly (approximately 30 seconds) with 1% (w/v) crystal violet (Sigma) in 20% (v/v) ethanol (EtOH, Sigma). Concentrations were normalised to tissue mass (in grams) or serum volume (in ml) as appropriate.

### Free oligosaccharide analysis

MDMΦ FOS were detected as previously described by Alonzi *et al*. [22]. Cells were cultured in 6-well plates as described above, and protein lysates were prepared according to the freeze/thaw protocol. Following cell lysis, samples were subjected to mixed-bed ion exchange and then lyophilised. FOS were labelled with 2-aminobenzoic acid (2-AA) and purified using a DPA-6S column (Sigma). Unconjugated 2-AA was removed by phase splitting with ethyl acetate, and samples were lyophilised and resuspended in water and then purified using a con-A column. Glycans were separated by normal phase-high performance liquid chromatography (NP-HPLC), and peak area was used to assess molar quantity in comparison to standards of known identity and quantity. FOS generation was normalised to protein concentration based on a modified Bradford assay as previously described [35]. Briefly, cell lysates were added in a 1:1 volumetric ratio to 1x Bradford Quick Start Reagent (Bio-Rad) and incubated at 20°C for 5 minutes. Absorbance values (A_xxx_) at 595 nm and 420 nm were measured, and the ratio of A_595_ to A_420_ was used to determine protein concentration in comparison to a serially diluted standard of bovine serum albumin.

### Glycosphingolipid analysis

MDMΦ glycosphingolipids (GSLs) were detected using NP-HPLC as previously described [36]. Briefly, cells were cultured in TC-25 flasks at 1.5 x 10^6^ cells/ml as described above and treated with appropriate dilutions of iminosugars for 48 hours at 37°C, 5% CO_2_. Following incubation, supernatants were removed and cells were washed twice in pre-warmed PBS before mechanical removal by scraping. Flasks were washed once with warm PBS, and this wash was combined with the scraped cell fraction and pelleted by centrifugation (room temperature, 4000 x *g*, 5 minutes). The MDMΦ pellet was washed once more in PBS before resuspension in 300 μl of water. Three cycles of freeze/thawing were executed to lyse samples, and an aliquot was removed for modified Bradford protein assay for normalisation. The remaining fraction was used to extract glycolipids by adding 400 μl chloroform and 800 μl methanol (MeOH) to 300 μl of aqueous lysate for lipid extraction using a modified Svennerholm and Fredman method [37]. Extracted GSLs were hydrolysed overnight by incubation with ceramide glycanase (37°C, 50 mM sodium acetate buffer, *p*H 5.0, containing 1 mg/ml sodium taurodeoxycholate). Oligosaccharides released from GSLs were brought to 30 μl with water and labelled with 2-AA as described for FOS. These labelled oligosaccharides were analysed by NP-HPLC as described above.

### Viral RNA quantification

DENV RNA in cell culture supernatants was isolated according to the manufacturer’s protocol for Direct-zol RNA MiniPrep Kit (Zymo Research) and assayed by reverse transcription-real time polymerase chain reaction (qRT-PCR) on an Applied Biosystems 7500 real-time PCR system (Life Technologies). Thermo-Start DNA Taq polymerase was used to enable Taq-mediated release of fluorescently labelled dyes from a DENV2 NS5-specific probe adapted from a previously published protocol [38]. Probe and primer sequence and concentrations were as previously described, with the reaction mixture prepared according to the manufacturer’s instructions for Verso 1-Step RT-PCR Kit with Thermo-Start Taq (Life Technologies). Thermal cycling was adapted to match enzyme components as follows. Synthesis of complementary DNA (cDNA) was performed for 30 minutes at 50°C, followed by a 15-minute activation of Thermo-Start *Taq* polymerase at 95°C. PCR thermocycling with fluorescence detection was executed for 45 cycles of 95°C for 15 seconds followed by 60°C for 60 seconds and a fluorescence read step. Samples were read in technical duplicate and compared to a standard curve generated from high-titer viral RNA isolated from C6/36-grown DENV2. 95% confidence intervals were determined based on biological and technical variation and graphed using Prism 6 (GraphPad Software, Inc.).

For animal experiments, multiplex qRT-PCR was conducted on all tissue isolates and plasma as described for *in vitro* experiments. Rodent glyceraldehyde 3-phosphate dehydrogenase (GAPDH) control (Life Technologies #4308313) containing a VIC reporter dye was attached to a TAMRA (tetramethylrhodamine) quencher, and experimental DENV NS5-specific target was labelled with FAM (6-fluorescein amidite) reporter dyes with a TAMRA quencher. GAPDH primers were used at 10 μM and probe at 20 μM. In the case of DENV qRT-PCR, quantitation using a DENV2-specific primer/probe set directed to NS5 was conducted using all primers and probe at 10 μM final concentration [38]. Thermocycling was conducted as for *in vitro* experiments. Viral RNA from tissues was quantified by ΔΔCt method, whereas plasma samples were quantified relative to a standard curve as for *in vitro* experiments. Samples were read in technical duplicate and 95% confidence intervals were determined based on biological and technical variation and graphed using Prism 6 (GraphPad Software, Inc.).

### DENV infection of mice and antiviral treatment

All experimental procedures were pre-approved by the UC Berkeley Animal Care and Use Committee and were performed according to the guidelines of the UC Berkeley Animal Care and Use Committee, the United States Public Health Service, and the USDA Animal Welfare Act. Mice of the 129/Sv background deficient in interferon (IFN)-α/β and -γ receptors (AG129 mice) were infected with DENV under antibody-enhanced conditions as previously described [5,39]. Mice were primed 24 hours prior to infection with a 5 μg intraperitoneal (i.p.) injection of a pan-flavivirus monoclonal antibody against E protein, 4G2. At t = 0, 10^5^ pfu of DENV2 D2S10 was injected intravenously. Beginning immediately following infection, mice were treated with 33.3 mg/kg celgosivir three times daily (t.i.d.) via oral gavage (p.o.). Treatment was continued for 80 hours, at which point mice were anaesthetised by isoflurane inhalation and euthanized after cardiac puncture.

Cardiac bleeds (approximately 1 ml) were collected in citrate anti-coagulant tubes (Becton Dickinson) and stored on ice prior to isolation of PBMCs and plasma. To separate plasma, whole blood was centrifuged (18,000 x *g*, 30 minutes, 4°C), and supernatant was collected. Cells were resuspended in 0.14 M ammonium chloride (Sigma), 17 mM Tris (MP Biomedicals), *p*H 7.2 in H_2_O and incubated at 37°C for 5 minutes to lyse red blood cells (RBCs). RBC lysis was repeated three times, and cells were washed in serum-free α-MEM (Gibco) prior to resuspension and storage in RNAlater (Qiagen). Spleen, liver, small intestine, kidney, and lymph node tissues were collected into tubes containing zirconia/silica beads (1.0 mm diameter) and protease inhibitor cocktail (Roche) in α-MEM. Tissues were homogenised using a Mini-Beadbeater-8 (BioSpec Products) and stored immediately at -80°C until future use. PBMCs and other tissue samples were thawed on ice in the presence of 40 mM dithiothreitol (DTT) and mixed with three volumes of buffer RLT (Qiagen) containing 40 mM DTT prior to isolation of cellular RNA by RNeasy Mini Kit (Qiagen) as per the manufacturer’s instructions. Plasma RNA (viral RNA) was isolated by QIAamp Viral RNA Mini Kit (Qiagen) in accordance with the manufacturer’s instructions.

### Statistical analyses

Antiviral EC_50_s in cell culture were calculated using a 4-parameter logistic model to obtain 95% confidence intervals in GraphPad Prism 6 (GraphPad Software, Inc.). The same protocol was applied to GSL assays. Statistically significant differences (α<0.05) in viral load for animal studies were assessed by non-parametric Mann-Whitney testing with Bonferroni correction for multiple comparisons.

## Results

### Determination of antiviral efficacy of iminosugars in primary human MDMΦs

**Fig 2:**
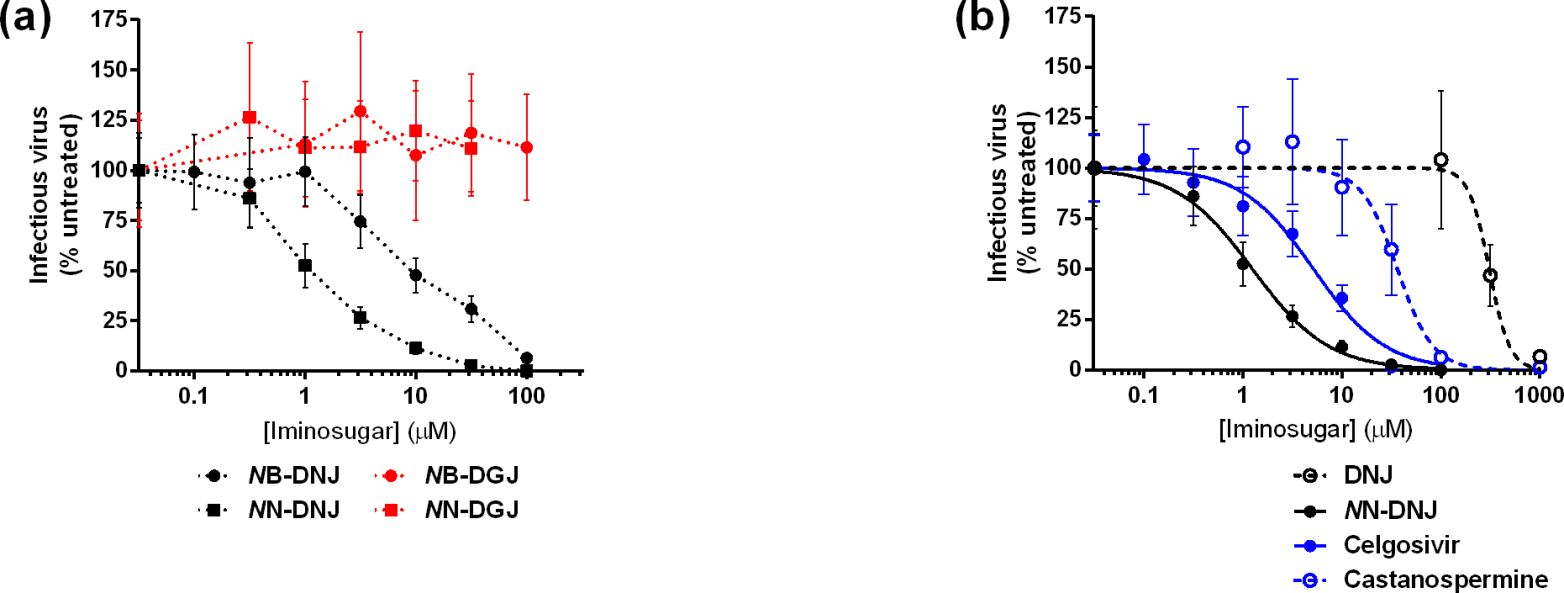
*In vitro* evaluation of iminosugar antiviral efficacy against DENV. MDMΦs were infected with DENV (serotype 2, MOI = 1) and treated with a titration of iminosugars for 48 hours. Infectious virus titer was determined by plaque assay using LLC-MK2 (monkey kidney) cells. Counts were normalised to 100 percent for untreated samples. **(a)** DNJ-derived iminosugars (black) had antiviral efficacy with EC_50_s between 1.2 and 10.6 μM, whereas DGJ-derived iminosugars (red) were not effective up to the maximum non-toxic dose tested. **(b)** Parent compounds DNJ and castanospermine were compared to derivatives *N*N-DNJ and celgosivir, respectively. A four-parameter logistic nonlinear regression curve was determined to calculate EC_50_ values. Enhancement of antiviral activity by derivatization for *N*N-DNJ was 246 fold and for celgosivir 7 fold. Each donor was treated in triplicate, and plaque assays were conducted in triplicate on each sample obtained. A minimum of three donors were tested for each compound. Data are presented as mean ± SD.

Cells of the myeloid lineage, including tissue-resident macrophages and dendritic cells as well as circulating monocytes, are instrumental in supporting early DENV infection and directing host immune responses [40–42]. We have previously established a monocyte-derived macrophage (MDMΦ) model using blood from dengue-naïve human donors [31] and demonstrated that iminosugars such as *N*B-DNJ and *N*N-DNJ can reduce production of infectious DENV in this model [5]. In order to dissect how these iminosugars reduce infectious virus, we first compared the antiviral activity of DNJ-derived and DGJ-derived compounds. MDMΦs were infected with DENV serotype 2 at an MOI of 1 for 90 minutes prior to treatment with drug. Upon removal of virus, a titration of iminosugar was added to the cells. At 48 hours post-infection, supernatants were collected and plaqued on LLC-MK2 cells. The relative titer of treated samples is presented as a percentage of the infectious titer of samples not treated with iminosugar (untreated) because of the variation observed in the titer of untreated samples from different donors (see S1 Table). IL-4-treated primary human macrophages produce an average of [2.58 +/- 2.07] x 10^4^ pfu/ml (mean +/- SD) of infectious DENV 48 hours after infection with MOI 1 DENV2, strain 16681, covering a >40-fold range from 2.3–97.5 x 10^4^ pfu/ml. DNJ-derived compounds successfully reduced production of infectious virus with EC_50_s between 1.2 and 10.6 μM, as we have previously reported [5]; however, there was no detectable reduction of infectious virus with any DGJ-derived compound up to the maximum non-toxic dose tested (Fig 2A). Cytotoxicity as assessed by MTS assay was not detected up to 100 μM for *N*B-DGJ, but for *N*N-DGJ the maximum non-toxic dose was determined to be 31.6 μM by MTS assay.

Although celgosivir has been tested previously in cell culture and animal models, the drug’s efficacy against DENV has not been assessed in primary human cells cultured *in vitro*. Furthermore, the antiviral properties of the pro-drug in comparison to castanospermine have not been addressed. In order to compare the optimization efforts of monocyclic and bicyclic iminosugars, we performed a titration of *N*N-DNJ and celgosivir as well as the “parent” compounds DNJ and castanospermine in our human MDMΦ model. Comparison of the two optimized candidates in MDMΦs reveals similar antiviral efficacy (EC_50_ *N*N-DNJ = 1.25 μM; EC_50_ celgosivir = 5.17 μM), and both drugs are at least 7-fold more potent than the parent compounds (Fig 2B). A summary of *in vitro* antiviral properties for all iminosugars tested is presented in Table 1.

**Table 1:** Summary of *in vitro* antiviral properties of iminosugars.

Stereochemistry	Class	Drug	EC_50_^a^ (μM)	MNTD^b^ (μM)	Enhancement^c^ (fold)
Glucose	DNJ	DNJ	308 (273–347)	10000	n.d.^d^
		*N*B-DNJ	10.6 (7.77–14.5)	100	29
		*N*N-DNJ	1.25 (1.05–1.48)	31.6	246
	Castanospermine	Castanospermine	36.4 (26.0–51.0)	100	n.d.
		Celgosivir	5.17 (3.69–7.24)	100	7
Galactose	DGJ	*N*B-DGJ	n.d.	100	n.d.
		*N*N-DGJ	n.d.	31.6	n.d.

^a^EC50 is given as the estimated value with 95% confidence interval in parentheses.

^b^MNTD is the maximum non-toxic dose tested.

^c^Enhancement is the fold improvement of antiviral EC_50_ for the compound of interest in comparison to the parent compound (e.g. EC_50 DNJ_/EC_50 NB-DNJ_ = 29).

^d^n.d. indicates that a value could not be determined.

### Evaluation of *in vitro* Enzyme Inhibition Profiles of DNJ and DGJ compounds

Given the diversity of functions of glycans *in vivo* and the rationale of iminosugar use as monosaccharide mimetics, such drugs might inhibit a considerable number of enzymes involved with glycan processing. Indeed, the differential antiviral effects of gluco- and galactostereochemistry-possessing iminosugars indicate that differential inhibition of molecular targets occurs in response to small, defined changes in stereochemistry. With alteration of one stereocenter, differences in inhibition of glucosidase and glycosidase enzymes specific for various sugar stereochemistries are likely. Such differences were hypothesized to account for the differential antiviral efficacy observed in cell culture. In order to address this possibility, a panel of isolated glycan processing enzymes were treated with the iminosugars used in this study to determine targets of inhibition (Table 2). Unsurprisingly, DNJ and derivatives thereof demonstrated high nanomolar to low micromolar *in vitro* inhibition of α-glucosidases, whereas DGJ-derived iminosugars only inhibited these enzymes at concentrations approaching 1 mM or not at all. Castanospermine and the pro-drug, celgosivir, were similarly capable of inhibiting the α-glucosidases; however, inhibition of mouse ER α-glucosidase II required considerably higher concentrations of drug than for the DNJs. Stereochemistry is similarly important for inhibition of α-galactosidase, as this enzyme is inhibited at low nanomolar concentrations by the DGJ iminosugars with identical stereochemistry to the natural substrate galactose, but absence of or very weak inhibition was detected for the glucose-mimetics. Of particular interest is the trend observed for inhibition of glucosyltransferase isolated from a human promyelocytic leukemia cell line (HL60) where iminosugars with increasing alkyl chain length, again mimicking the natural substrate of the enzyme glucosylceramide synthase, were more potent inhibitors of the enzyme. A similar trend can be observed for both DNJ and DGJ iminosugars, suggesting that this effect is not specific to sugar stereochemistry.

**Table 2:** *In vitro* enzyme inhibition of iminosugars.

			IC_50_^a^ (μM)						
Class	Enzyme	DNJ	*N*B-DNJ	*N*N-DNJ	DGJ	*N*B-DGJ	*N*N-DGJ	Castanospermine	Celgosivir
α-glucosidase	Mouse ER α-glucosidase II	11.4	5.2	3.1	>1000	>1000	>1000	58.9	158.1
	Rat intestinal maltase	0.2	1.1	0.46	362	>1000	>1000	0.37	17
	Rat intestinal isomaltase	0.8	2.1	7.3	>1000	>1000	>1000	6.1	29.3
	Rat intestinal sucrase	0.51	0.44	0.74	340	>1000	>1000	0.28	2.7
	Human lysosome	0.37	0.27	0.14	>1000	>1000	>1000	2.0	93.2
Glucosyltransferase	HL60^b^	>1000	8.8	0.28	>1000	32.7	5.2	>1000	>1000
α-galactosidase	Human lysosome	217	>1000	765	0.06	3.1	35.5	>1000	>1000

^a^IC_50_ is the concentration required to inhibit the enzyme to 50% activity.

^b^HL60 is a human promyelocytic cell line from which the glucosyltransferase has been isolated.

### Iminosugars inhibit glycolipid processing in cell culture

**Fig 3:**
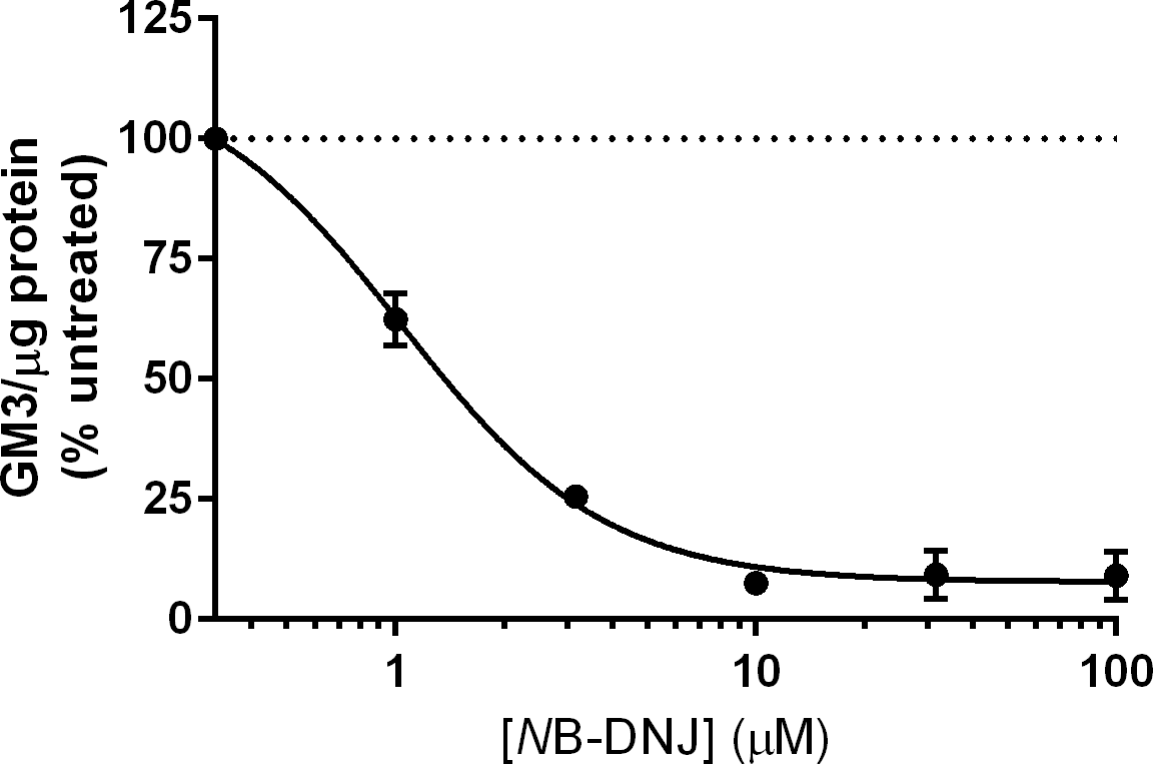
*N*B-DNJ reduces glycolipid production in a potent, dose-dependent manner in primary human MDMΦs. Uninfected MDMΦs were treated with a titration of *N*B-DNJ for 48 hours, and glycolipids were isolated from whole cell lysates. Production of monosialodihexosylganglioside (GM3) was normalized to total protein content for each sample, and each treatment was normalized to untreated controls on a donor-specific basis. *N*B-DNJ inhibited production of GM3 in a dose-dependent manner that was approximately 10-fold more potent than the antiviral inhibition. Data are presented as mean ± SD from assay of three biological replicates assayed in technical singlicate.

The *in vitro* testing of isolated enzymes indicates that inhibition of either α-glucosidases or glucosyltransferases is likely the essential property of iminosugars required for antiviral efficacy. Furthermore, the exclusive ability of glucose-mimicking iminosugars to potently inhibit α-glucosidases provides considerable support for the hypothesis that α-glucosidase inhibition is the single class of target enzymes responsible for antiviral activity against DENV; however, it is equally possible that only DGJ-derived iminosugars fail to access glycolipid processing enzymes in cells. Thus, we next assessed whether these iminosugars could achieve inhibition of glycolipid processing in our human MDMΦ model. In order to address this question, MDMΦs were treated for 48 hours with the maximal non-toxic dose (MNTD) of drug used in antiviral studies as in Table 1. Whole cell lysates were then collected and assayed for monosialodihexosylganglioside (GM3) levels. Gangliosides are reliable markers of iminosugar-mediated alteration of glycolipid processing [25,43], and the relative simplicity of GM3, possessing one β-d-galactose and one β-d-glucose head group, facilitates analysis by normal phase high performance liquid chromatography (NP-HPLC). As shown in Table 3, DNJ and DGJ derivatives both effectively lowered GM3 levels normalized to total protein concentration by at least 90 percent. In contrast, bicyclic glucose iminosugars failed to inhibit GM3 synthesis. In addition to inhibition at maximal dosing, the dose-response relationship of iminosugar to GM3 levels was investigated to determine whether this correlated with antiviral efficacy. The response of GM3 production in MDMΦs to *N*B-DNJ was therefore investigated in three donors over a titration of 1 to 100 μM of drug. Fig 3 shows that treatment resulted in a dose-dependent decrease in GM3 production. The EC_50_ of *N*B-DNJ with respect to reduction of GM3 is approximately 10-fold lower than the antiviral EC_50_ (95% CI for GM3 EC_50_ = 0.80–1.37 μM; 95% CI for antiviral EC_50_ = 7.77–14.5 μM). Although 90–95% inhibition of GM3 levels could be reached with 10 μM treatment of *N*B-DNJ, increasing concentrations up to 100 μM did not lead to further inhibition of GM3 levels, suggesting that this is the maximal effect possible for iminosugars. In summary, these data indicate that all of the monocyclic iminosugars tested could effectively inhibit glycolipid processing to equivalent levels at the concentrations tested in the antiviral assays presented in Fig 2A.

**Table 3:** GM3 inhibition in MDMΦ by treatment with MNTD iminosugar concentrations.

Drug	Concentration (μM)	GM3 production (%)^a^
DMSO (control)	0.06% (v/v)^b^	97.3 ± 3.7
*N*B-DNJ	100	8.8 ± 3.6
*N*B-DGJ	100	7.3 ± 1.9
*N*N-DNJ	31.6	5.9 ± 1.2
*N*N-DGJ	31.6	4.9 ± 1.9
Castanospermine	100	101.6 ± 6.7
Celgosivir	100	100.9 ± 4.1

^a^% is normalized to untreated controls for each donor and the value is presented as mean ± SD from a minimum of 3 donors.

^b^v/v indicates the volumetric ratio of DMSO to media used as a negative control to account for equivalent dilution of nonyl-derivative iminosugars for which stocks are made in DMSO rather than PBS.

### DNJ iminosugars inhibit glycoprotein processing in cell culture

**Fig 4:**
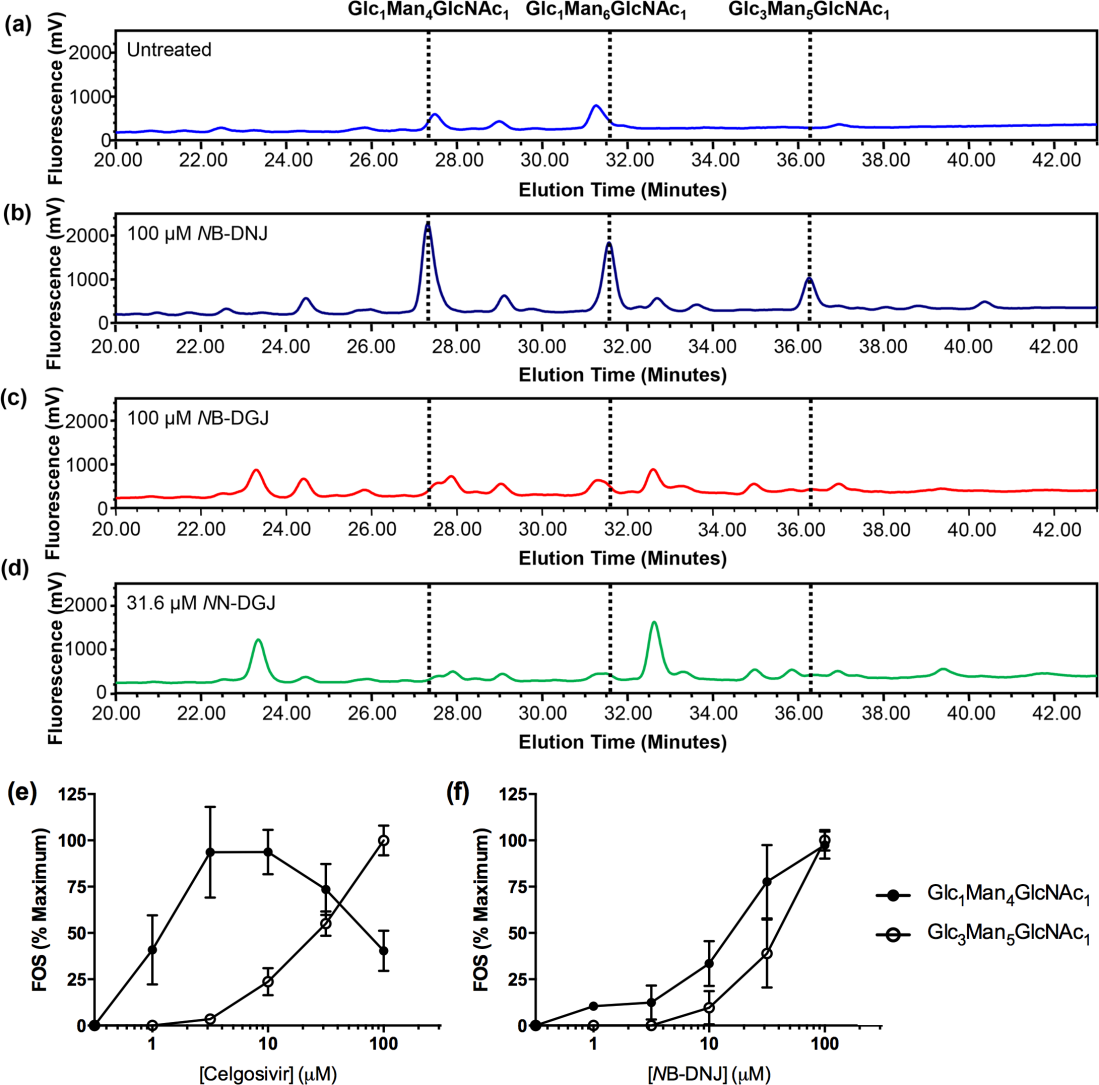
Iminosugars possessing glucostereochemistry but not galactostereochemistry produce ER α-glucosidase associated FOS in MDMΦs. MDMΦs were treated in technical duplicate for 48 hours with iminosugars. **(a)** Untreated cells do not produce detectable FOS representative of ER α-glucosidase inhibition. **(b)** Cells treated with 100 μM *N*B-DNJ iminosugar produce FOS representative of ER α-glucosidase I inhibition (Glc_3_Man_5_GlcNAc_1_) and ER α-glucosidase II inhibition (Glc_1_Man_4_GlcNAc_1_ and Glc_1_Man_6_GlcNAc_1_). **(c-e)** *N*B-DGJ **(c)**, and *N*N-DGJ **(d)** all fail to produce detectable ER α-glucosidase associated FOS, but do produce FOS generated by inhibition of lysosomal β-*N*-acetylhexosaminidase. **(e-f)** MDMΦs were treated with a serial dilution of celgosivir (**e**) or *N*B-DNJ (**f**), and inhibition of α-glucosidase I was measured by accumulation of Glc_3_Man_5_GlcNAc_1_ (white circles) while inhibition of α-glucosidase II was measured by accumulation of Glc_1_Man_4_GlcNAc_1_ (black circles). For each donor, the maximal concentration of each oligosaccharide species reached was normalized to 100%. Assays were conducted in technical duplicate on a minimum of three donors, of which one representative replicate is presented (**a-d**) or of which the mean ± SD is presented **(e-f)**.

Whereas both DNJ- and DGJ-derived iminosugars can effectively inhibit glycolipid processing in cell culture, the inhibition data from isolated enzymes suggests that only glucose-mimic iminosugars are likely to inhibit glycoprotein processing. Generation of FOS can be used as a marker of successful inhibition of ER-resident α-glucosidases [22], and this technique has previously been used to demonstrate inhibition of these glycoprotein processing enzymes by DNJ-iminosugars in a human lung cancer-derived cell line (A549) [23]. In order to address whether the iminosugars used in this study can also productively inhibit ER α-glucosidases in a primary human cell, more relevant for DENV infection, we treated our MDMΦ model with the MNTD of all iminosugars as well as a titration of celgosivir or *N*B-DNJ for 48 hours in the absence of DENV infection. As a consequence of inhibition of ER α-glucosidase II, a monoglucosylated glycoprotein is produced. After trimming of the glycan precursor by mannosidases and recognition of the glycoprotein as terminally misfolded, a Glc_1_Man_4_GlcNAc_1_ free oligosaccharide species is cleaved from the peptide during ER-associated degradation. In the case of inhibition of ER α-glucosidase I, a similar process produces a Glc_3_Man_5_GlcNAc_1_ species. Thus, the presence of each species of FOS can be correlated with successful inhibition of the respective cellular α-glucosidase [22]. Nearly all species of FOS were undetectable in the absence of iminosugar treatment (Fig 4A); however, addition of 100 μM *N*B-DNJ led to accumulation of peaks representative of inhibition of both α-glucosidases (Fig 4B). Similar inhibition of both α-glucosidases was also noted for *N*N-DNJ at the MNTD; however, all DGJ-derivatives tested failed to produce any detectable monoglucosylated or triglucosylated oligosaccharide species, which could be a product of inhibited glycoprotein processing at the MNTD (Fig 4C and 4D). The major peaks observed in the NP-HPLC trace for DGJ-treated samples, which occur at 23.3 and 32.6 minutes, represent FOS generated by inhibition of lysosomal β-*N*-acetylhexosaminidases [44]. These enzymes are responsible for trimming terminal glycans from molecules possessing terminal *N*-acetyl hexosamines (e.g. GlcNAc) and are frequently implicated in disorders of glycolipid processing [45].

Analysis of the dose-response relationship of iminosugars and FOS generation may provide further insights into the mechanism of action of the drugs. Addition of 1 μM *N*B-DNJ or celgosivir led to accumulation of Glc_1_Man_4_GlcNAc_1_ but not Glc_3_Man_5_GlcNAc_1_ in all donors tested (Fig 4E and 4F). With increasing concentrations of celgosivir, further accumulation of monoglucosylated FOS was observed, with maximal levels achieved at 3 and 10 μM drug. Whereas triglucosylated FOS were produced in relatively limited amounts at treatment below 10 μM, increasing accumulation of Glc_3_Man_5_GlcNAc_1_ was noted with 31.6 and 100 μM celgosivir treatment. A concomitant decrease in Glc_1_Man_4_GlcNAc_1_ accumulation was reliably detected, with the more prolific accumulation of triglucosylated FOS at the highest dose tested. Reduced accumulation of monoglucosylated FOS is presumed to be a function of substrate limitation as α-glucosidase I activity occurs upstream of α-glucosidase II activity; thus, inhibition of α-glucosidase I limits the available pool of substrate for α-glucosidase II. A similar trend to celgosivir was observed for *N*B-DNJ, except that the difference in inhibition of α-glucosidase I and α-glucosidase II was considerably less pronounced for *N*B-DNJ (Fig 4F). These differences in specificity may be a useful starting point for understanding glucosidase-specific structure-activity relationships of iminosugars. With a growing understanding of the specificity of different drugs in this class, it may be possible to ascertain the relative importance of inhibition of each α-glucosidase. Thus, although glycolipid inhibition fails to differentiate antiviral from non-antiviral iminosugars, glycoprotein inhibition correlates with antiviral activity.

### Iminosugar inhibition of α-glucosidases prevents secretion of virus

**Fig 5:**
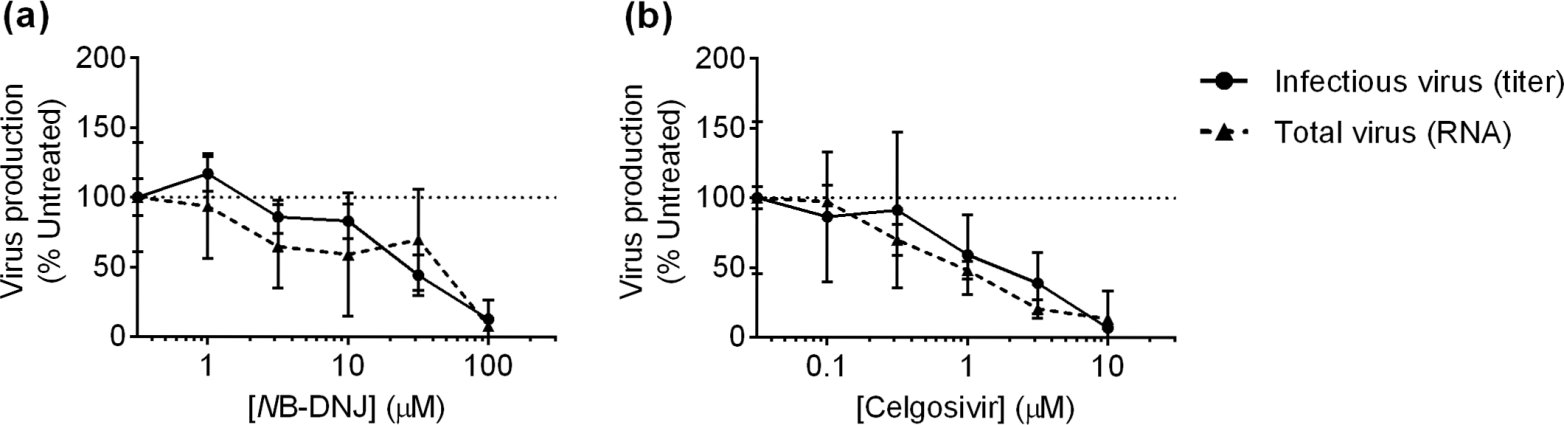
Glucostereochemistry iminosugars reduce secretion of DENV. MDMΦs were treated in technical triplicate for 48 hours with a serial dilution of iminosugars. Treatment with *N*B-DNJ (**a**), and celgosivir (**b**) reduces infectious virus (solid lines) and total virus (dashed lines) secreted in a one-to-one ratio. The values for both RNA and infectious virus are means normalized to untreated samples within a donor. Each donor was treated in technical triplicate and qRT-PCR was conducted in technical duplicate, while LLC-MK2 plaque assays were conducted on nine technical replicates. A single representative donor is plotted for each drug as mean ± SD.

Strict dependence of DENV glycoproteins on α-glucosidase trimming and subsequent interactions of the glycoproteins with the ER chaperones calnexin and/or calreticulin would explain the historical data as well as our observations of the antiviral efficacy of iminosugars against DENV. The sum of these historical data and the data presented above still fails to demonstrate whether the glycoproteins, particularly E, are grossly misfolded and targeted for degradation or whether partially misfolded glycoproteins escape the folding control of the ER, and the virus that subsequently buds from the cell is less infectious due to this partial misfolding, as seen for example in the case of iminosugar-treated HIV-secreting cells [46]. In order to address this question, we infected MDMΦs with DENV as in Fig 2 and treated them with a serial dilution of *N*B-DNJ or celgosivir. Supernatants were collected at 48 hours post-infection for coordinated assay of infectious virus by plaque assay and total virus production by qRT-PCR for DENV RNA. For both drugs and in every donor tested, the drop in infectious viral titer correlated with a drop in total virus secreted (Fig 5). To ensure that the reduction in total virus secretion observed at 48 hours was not related to re-infection of MDMΦs following a round of DENV replication within the cells (i.e. related to decreased infectivity), total DENV secretion levels at 24 hours were assayed and compared to those observed at 48 hours post-infection. Infectious DENV is only detectable in our MDMΦ model with incubation of greater than 12 hours as demonstrated in other cell systems [47,48]; thus, assays conducted at 24 hours are likely to be exclusively the product of events occurring only as a result of a single round of infection. In summary, the direct correlation of effects on total virus and infectious virus further supports the prevailing hypothesis that glucostereochemistry iminosugars, as a class, induce gross misfolding of DENV glycoproteins leading to ER-associated degradation and reduced secretion of virus.

### *In vivo* efficacy of bicyclic iminosugars

**Fig 6:**
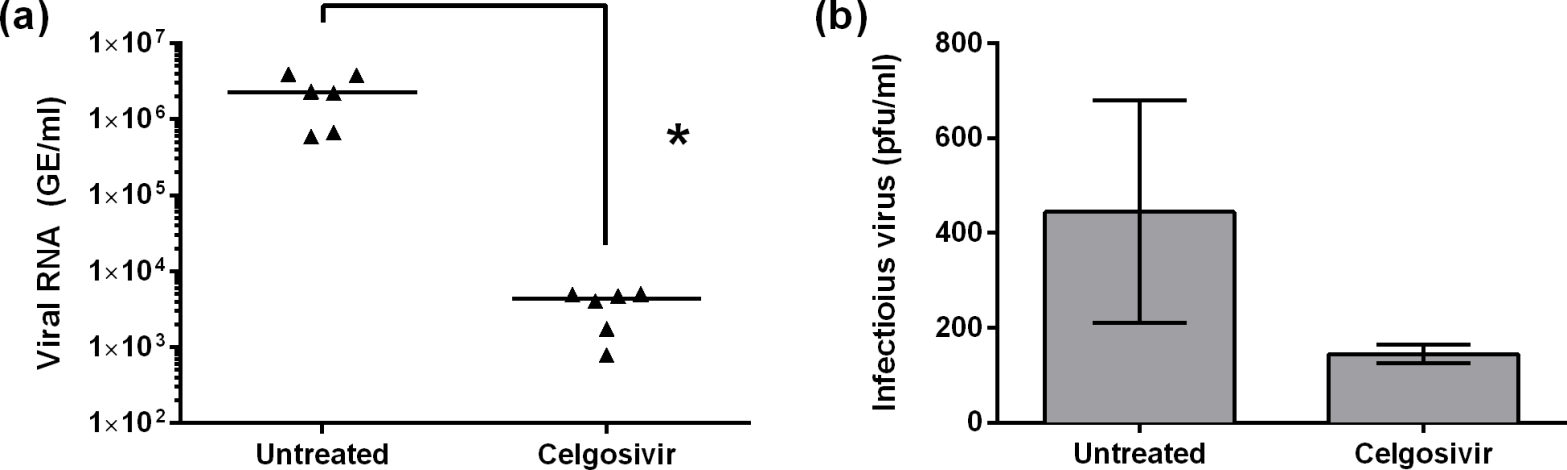
Celgosivir reduces circulating virus *in vivo* immediately prior to normal time of death in untreated controls. Iminosugar was given orally t.i.d. (celgosivir: 33.3 mg/kg/dose) in a lethal mouse model of antibody-enhanced DENV infection. Serum samples from terminal bleeds were collected from all mice 8 hours prior to normal time of death in infected, untreated controls. **(a)** Circulating viral load was significantly reduced by celgosivir treatment as assayed by qRT-PCR. Statistically significant differences (*, α<0.05) were assessed by non-parametric Mann-Whitney test with Bonferroni correction for multiple comparisons of RNA (see S1 Fig). Individual readings are plotted (triangles), with the median value for each treatment indicated by the horizontal line. **(b)** Infectious virus level in the same serum samples was assayed by BHK-21 plaque assay. Celgosivir reduced the average viral titer, but the differences were not statistically significant by ANOVA. Data are presented as mean ± SD. Three mice were tested for each compound, and both assays were conducted in technical duplicate.

Previous work has demonstrated that orally administered iminosugars can rescue mice infected with a lethal dose of DENV in models of severe disease [16–18,49]. Dosing of the iminosugars used in these studies has varied from once daily (QD) to thrice daily (t.i.d.) regimes with more frequent dosing generally associated with greater success. Although celgosivir has demonstrated efficacy at twice daily (b.i.d.) intraperitoneal (i.p.) dosing of 50 mg/kg/dose, recent human trials were conducted with an oral (p.o.) regime of a 400 mg loading dose followed by 200 mg/dose b.i.d. These trials failed to demonstrate reduction in viral load or fever burden in patients with dengue infection [50,51]. To address these inconsistencies and assess whether more frequent dosing with less drug might be more efficacious, we treated animals with 33 mg/kg/dose of celgosivir t.i.d. p.o. and assessed levels of viral RNA in circulation (Fig 6A) and in various tissues (S1 Fig) as well as infectious virus in circulation (Fig 6B). AG129 mice were administered 5 μg of 4G2 antibody i.p. 24 hours prior to infection with 10^5^ pfu of DENV-2 D2S10 to mimic ADE infection [5,39]. Drug was administered immediately following infection and every 8 hours thereafter until mice were sacrificed at 80 hours post-infection. In this mouse model of DENV disease, mortality occurs at day 3.5 to 4 (84–96 hrs) post-infection; thus, samples were collected just prior to sacrifice at a point when differences in variables anticipated to be relevant to clinical outcomes were predicted to be most pronounced. Serum was isolated from whole blood and used to determine the viral load (circulating viral RNA, Fig 6A) and the level of infectious virus (Fig 6B).

Using this more frequent, oral treatment regime for celgosivir, there was a statistically significant reduction in viral RNA load (α<0.05), and this trend appeared to extend to infectious virus; however, results for the plaque assay were not statistically significant (α = 0.100) due to the considerable variation in the assay. Additional tissues including kidney, parenteral lymph nodes, liver, and small intestine showed similar trends in RNA levels; however, in the case of the spleen, celgosivir enhanced viral RNA levels by approximately two orders of magnitude (S1 Fig). Overall, these results indicate that more frequent oral dosing with less drug administered per dose may be a viable antiviral treatment strategy for celgosivir. As celgosivir does not inhibit the glycolipid biosynthesis pathway (Table 3), any antiviral effect observed here is due to ER α-glucosidase inhibition.

## Discussion

In this work, we tested a panel of iminosugars covering a range of specificities of enzyme inhibition profiles to address the role of glycoprotein and glycolipid inhibition on DENV antiviral activity. Coordinated analysis of isolated enzyme inhibition profiles, primary human cell culture antiviral, glycoprotein, and glycolipid inhibition profiles, and animal model efficacy allows us to present the most complete deconstruction of iminosugar antiviral properties necessary for efficacy against DENV to date. Prior to this publication, a large body of work existed in support of the hypothesis that iminosugar antiviral activity against DENV is a function of inhibition of endoplasmic reticulum resident α-glucosidases; however, exclusion of alternative mechanisms of action related to inhibition of additional sugar processing enzymes had not been undertaken. Knowledge of the precise mechanism(s) of action of iminosugars as DENV therapeutics is essential as these drugs enter and progress through clinical trials.

The possibility that some level of antiviral activity is a function of modulation of alternative targets including host glycolipid processing and/or viral ion channel inhibition bears precedent [52–55]. There is some debate as to the existence of an ion channel in DENV [56,57] although the best available evidence suggests that there is no equivalent molecule to the HCV ion channel p7 [56]. Iminosugars are also known to inhibit glycan processing enzymes in addition to ER α-glucosidases. Reports of inhibition of intestinal disaccharidases [58] were confirmed for both *N*B-DNJ and celgosivir (Table 2); however, we cannot conceive of a mechanism whereby this effect on enzymes located on the brush border of the small intestine could accomplish antiviral activity in the experimental setting investigated here, or indeed when iminosugars are administered systemically. To our current knowledge, there is no evidence for an antiviral mechanism mediated by inhibition of additional human glycosidases. In contrast, the role of glycolipids in viral genesis provides support for the hypothesis that this “off-target” effect may play a role in iminosugar antiviral activity. The ability of iminosugars to inhibit glycolipid processing as in the clinical use of *N*B-DNJ for Gaucher’s disease and Niemann-Pick type C disease [24,25,59] provides cause for concern that the promiscuity of iminosugars for multiple glycan processing enzymes has clouded our understanding of antiviral drug design and optimization.

Our results demonstrate that production of infectious DENV in a primary human macrophage model was controlled in a dose-dependent manner with DNJ-derivative iminosugar treatment, as previously reported [5], and variable alkylation of the ring nitrogen enhanced antiviral activity. A similar inhibition profile was noted in MDMΦs for *N*N-DNJ and celgosivir, although the improvement of *N*N-DNJ in comparison to the parent compound DNJ (246-fold) was considerably more pronounced than the improvement of celgosivir in comparison to the parent castanospermine (7-fold). In a BHK-21 (hamster kidney) cell culture model, celgosivir exhibited ~100-fold enhancement of antiviral efficacy over castanospermine [17]; however, this difference in enhancement may be due to variable efficiency of cleavage of the butanoyl pro-drug moiety in different cell culture models. Strikingly, equivalent galactostereochemistry analogues of the DNJ derivatives all failed to impact infectious virus production in MDMΦs suggesting a clear importance for the stereochemistry of the iminosugar head group.

How does the difference in glucose and galactose stereochemistry confer such a striking difference to antiviral efficacy of iminosugars? Although it remains a technical challenge to produce sufficient quantities of pure human ER α-glucosidase to test this enzyme *in vitro*, our panel of closely related enzymes helps to answer the question of iminosugar specificity. Inhibition of isolated enzymes occurs in line with anticipated headgroup stereospecificity (e.g. galactose analogues inhibit galactosidases and not glucosidases), but this specificity does not extend to glycolipid processing enzymes such as the glucosyltransferase of HL60 cells, where factors relating to alkyl chain structure and length are more likely to be relevant. It is worth noting that the inhibition of enzymes *in vitro* is less potent for celgosivir than for castanospermine, a result that is likely to be the product of interference of the butanoyl group with binding of the inhibitor. In cell culture and animal models, this group is cleaved so that the more potent inhibitor, castanospermine, is in abundance. Trends observed for isolated enzymes are replicated in cell culture; thus, the question of antiviral efficacy cannot be explained by differential uptake, a phenomenon much more likely to be mediated by differential alkylation. Rather, this work demonstrates that in the presence of maximal glycolipid processing inhibition (~95% reduction of GM3), DGJ-derived iminosugars fail to induce any accumulation of FOS related to ER α-glucosidase inhibition. Although equivalent glycolipid processing effects are observed for DNJ-derivatives, the cellular inhibition of ER α-glucosidases correlates remarkably well with observed antiviral efficacy of iminosugars. Whereas GM3 accumulation occurs at approximately 10-fold lower concentrations, α-glucosidase inhibition maps very well to antiviral efficacy. In particular, inhibition of α-glucosidase II appears to be of specific importance as can be observed by comparison of celgosivir and *N*B-DNJ titration curves which reveals a more potent inhibition of α-glucosidase II by the more potent antiviral, celgosivir. This trend can be extended even in the case where α-glucosidase I inhibition occurs at lower concentrations than α-glucosidase II inhibition. In the case of DNJ based iminosugars CM-10-18 and CM-9-78, α-glucosidase I inhibition occurs at 3- to 5-fold lower concentrations than α-glucosidase II concentrations. For these molecules, α-glucosidase II inhibition occurs with an EC_50_ of 2.6 and 1.6 μM for CM-9-78 and CM-10-18, respectively. Antiviral EC_50_s in the same A549 cell line are 1.5 and 1.1 μM while α-glucosidase I inhibition EC50s of 0.42 and 0.46 μM are noted, respectively [23]. Thus, we propose that inhibition of α-glucosidase II is the crucial property of iminosugars for antiviral activity against DENV.

It has been suggested that iminosugars reduce infectivity of HIV [46,60] but prevent secretion of HBV [61]. In our hands, it appears that DENV secretion is blocked and the virus that does escape is similar in infectivity to virus produced in the absence of iminosugars, thus the effect that iminosugars have on DENV appears to be more similar to that of HBV rather than HIV. Previously published studies indicate that iminosugar treatment during the course of DENV infection reduces association of viral glycoproteins with the ER chaperones calnexin and calreticulin [6], and our ability to detect FOS that have been cleaved from terminally misfolded glycoproteins suggests that DENV glycoproteins are not retained by ER chaperones and are instead grossly misfolded and targeted for ER-associated degradation when cells are treated with iminosugars.

Lack of antiviral efficacy observed in the first human studies of iminosugars as DENV therapeutics is a challenge for the field [50,62]. Use of the AG129 ADE model of DENV infection is well established in antiviral drug testing, so we sought to address the events that occur with drug administration immediately prior to the normal time of death to capture any virus-related phenotype that may be responsible for protection with iminosugar treatment. This experiment demonstrated that more frequent dosing of celgosivir at lower doses than have been previously tested are capable of reducing levels of tissue-resident and circulating viral RNA. It should be noted that a pronounced level of circulating viral RNA and infectious virions could still be detected in animals treated with celgosivir. This suggests that complete elimination of virus by iminosugar treatment is not essential for survival, but reduced viral load and any concomitant changes in response to pathogen clearance are sufficient to prevent mortality. Furthermore, any viral RNA measured by qRT-PCR in the mouse model that is not accounted for by infectious virus theoretically represents viral RNA encapsulated in non-infectious virions. As such, comparison of the *in vitro* macrophage data in Fig 5 and *in vivo* mouse data in Fig 6 suggests that in the mouse model there is an accumulation of viral RNA encapsulated in non-infectious virions that is not observed in primary macrophage cell culture. The extension of these observations in a mouse model to the treatment of human infection requires further investigation, but this is consistent with studies demonstrating that viral load correlates with disease severity [51]. Finally, these data further support the hypothesis that glucosidase inhibition is sufficient for anti-DENV activity.

In summary, this study provides confirmation of the long-standing dogma that iminosugar inhibition of DENV is accomplished via inhibition of ER α-glucosidases. Our data are in agreement with the mounting bank of evidence that α-glucosidase II is the glucosidase responsible for anti-DENV activity, and we suggest that any further optimisation of iminosugar candidates focuses on enhanced inhibition of this enzyme. For monocyclic iminosugars, the necessary specificity for glucose-processing enzymes is provided by the DNJ headgroup. Alkylation or modification of the ring nitrogen on the other hand constitute amenable targets for medicinal chemistry efforts. Modulation of the alkyl group in particular is promising as we have demonstrated that glycolipid inhibition, which may be altered or abolished with changes in the alkyl group is of no consequence to antiviral activity against DENV. At present, high-resolution structural data for the complete α-glucosidase II enzyme is unavailable; however, a crystal structure of a lectin receptor domain has been solved [63]. Availability of more complete structural data for this enzyme would enable structure-activity relationship investigations of iminosugars and may provide insights to the differential inhibition observed in this study. Looking to the future, these data provide a rationale for further optimization of iminosugar structure and treatment regimes as dengue virus therapeutics.

## Supporting Information

S1 Fig Celgosivir affects levels of tissue-resident virus *in vivo* immediately prior to normal time of death in untreated controls. Tissues were collected from the mice treated in Fig 3 at the time of sacrifice, and viral load was determined by qRT-PCR. Viral load in genome equivalents was normalized to GAPDH (ng). Three mice were tested for each compound, and both assays were conducted in technical duplicate. Statistically significant differences (*, α<0.05) were assessed by non-parametric Mann-Whitney tests followed by Bonferroni correction for multiple comparisons. Individual readings are plotted (triangles) with the median value for each treatment indicated by the horizontal line. Organs assayed were: **(a)** parenteral lymph nodes, **(b)** small intestine, **(c)** liver, **(d)** kidney, and **(e)** spleen.
(DOCX)

S1 Table Variation in infectious titre of DENV released from primary human macrophages in the absence of drug treatment. Titre of infectious dengue virus measured in the supernatant of primary human MDMΦs 2 days after infection with MOI 1 DENV2, strain 16681, as determined by plaque assay. Cells were treated with 25ng/ml recombinant human IL-4 for 3 days prior to infection with DENV, as described in Methods.
(DOCX)
