# Supplementary material for: Iminosugars Inhibit Dengue Virus Production via Inhibition of ER Alpha-Glucosidases—Not Glycolipid Processing Enzymes
Source: PLoS Negl Trop Dis. 2016 Mar 14;10(3):e0004524. doi: 10.1371/journal.pntd.0004524 (PMC4790851; doi:10.1371/journal.pntd.0004524)
Supplement: S1 Fig — Tissues were collected from the mice treated in Fig 3 at the time of sacrifice, and viral load was determined by qRT-PCR. Viral load in genome equivalents was normalized to GAPDH (ng). Three mice were tested for each compound, and both assays were conducted in technical duplicate. Statistically significant differences (*, α<0.05) were assessed by non-parametric Mann-Whitney tests followed by Bonferroni correction for multiple comparisons. Individual readings are plotted (triangles) with the median value for each treatment indicated by the horizontal line. Organs assayed were: (a) parenteral lymph nodes, (b) small intestine, (c) liver, (d) kidney, and (e) spleen. (DOCX) [file pntd.0004524.s001.docx]

**

**

**S1 Fig. Celgosivir affects levels of tissue-resident virus *in vivo* immediately prior to normal time of death in untreated controls.** Tissues were collected from the mice treated in **Fig 3** at the time of sacrifice, and viral load was determined by qRT-PCR. Viral load in genome equivalents was normalized to GAPDH (ng). Three mice were tested for each compound, and both assays were conducted in technical duplicate. Statistically significant differences (*, α<0.05) were assessed by non-parametric Mann-Whitney tests followed by Bonferroni correction for multiple comparisons. Individual readings are plotted (triangles) with the median value for each treatment indicated by the horizontal line. Organs assayed were: **(a)** parenteral lymph nodes, **(b)** small intestine, **(c)** liver, **(d)** kidney, and **(e)** spleen.
