# Supplementary material for: Iminosugars Inhibit Dengue Virus Production via Inhibition of ER Alpha-Glucosidases—Not Glycolipid Processing Enzymes
Source: PLoS Negl Trop Dis. 2016 Mar 14;10(3):e0004524. doi: 10.1371/journal.pntd.0004524 (PMC4790851; doi:10.1371/journal.pntd.0004524)
Supplement: S1 Table — Titre of infectious dengue virus measured in the supernatant of primary human MDMΦs 2 days after infection with MOI 1 DENV2, strain 16681, as determined by plaque assay. Cells were treated with 25ng/ml recombinant human IL-4 for 3 days prior to infection with DENV, as described in Methods. (DOCX) [file pntd.0004524.s002.docx]

**S1 Table. Variation in infectious titre of DENV released from primary human macrophages in the absence of drug treatment.** Titre of infectious DENV measured in the supernatant of primary human MDMΦs 2 days after infection with MOI 1 DENV2, strain 16681, as determined by plaque assay. Cells were treated with 25ng/ml recombinant human IL-4 for 3 days prior to infection with DENV, as described in Methods.

| **Donor number** | **Titre in cell supernatant (pfu/ml)** |
| --- | --- |
| 1 | 19972 |
| 2 | 10433 |
| 3 | 2722 |
| 4 | 23111 |
| 5 | 2722 |
| 6 | 48556 |
| 7 | 22889 |
| 8 | 22556 |
| 9 | 22222 |
| 10 | 6111 |
| 11 | 13111 |
| 12 | 6111 |
| 13 | 25333 |
| 14 | 60667 |
| 15 | 40286 |
| 16 | 26556 |
| 17 | 22111 |
| 18 | 34222 |
| 19 | 19692 |
| 20 | 26222 |
| 21 | 2350 |
| 22 | 24111 |
| 23 | 97500 |
| 24 | 25000 |
| 25 | 39333 |
